# Cerebral Air Embolism Following Transthoracic Lung Biopsy Successfully Treated With Hyperbaric Oxygen

**DOI:** 10.7759/cureus.32933

**Published:** 2022-12-25

**Authors:** Mafalda Silva, Clara Gaio-Lima, Óscar Camacho, Joana Ribeiro

**Affiliations:** 1 Anesthesiology, Centro Hospitalar Vila Nova de Gaia/Espinho, Porto, PRT; 2 Hyperbaric Medicine, Hospital Pedro Hispano, Matosinhos, PRT; 3 Pulmonology, Unidade Local de Saúde da Guarda, Guarda, PRT

**Keywords:** emergency, undersea and hyperbaric medicine, hyperbaric oxygen treatment, cerebral air embolism, lung biopsy

## Abstract

Transthoracic lung biopsy is a frequently performed procedure performed worldwide. Although rare, air embolism is a potentially fatal complication. Rapid diagnosis and immediate treatment are essential to prevent patient clinical deterioration. Hyperbaric oxygen treatment is the standard of care in air embolism and time referral is critical for patient prognosis. We report a case of a man who underwent a percutaneous transthoracic lung biopsy which was complicated with arterial air embolism and severe neurologic symptoms; he was successfully treated with hyperbaric chamber treatment.

Physicians performing such techniques should be aware of this severe complication as immediate support treatment and hyperbaric oxygen may prevent irreversible neurologic lesions.

## Introduction

Percutaneous computerized tomography (CT) guided lung biopsy is a frequently performed procedure for pulmonary lesions and is often considered for peripheral lesions or when another procedure has failed to obtain diagnostic tissue for histological characterization [1]. The most frequently observed complications include pneumothorax (27%), intraparenchymal hemorrhage (11%), and hemoptysis (7%) which are usually treated conservatively [2].

Air embolism is a rare but potentially fatal complication of such techniques as the embolus may achieve cerebral or coronary circulation. Symptomatic air embolism was previously reported to have an incidence of 0.08% [1,3]. Air embolism is caused by the entrance of gas either into the pulmonary or systemic circulation [3]. A gas volume of only 2 mL may occlude functional cerebral arteries and be fatal [4].

Hyperbaric oxygen is the first-line therapy for arterial air embolism and should be initiated immediately when available [1]. Breathing 100% oxygen at a pressure above that of the atmosphere aims to reduce bubble size and prevent cerebral edema. Neurologic recovery and prognosis are dependent on time referral to hyperbaric treatment [3].

We report a case of a percutaneous transthoracic CT-guided lung biopsy complicated with cerebral arterial air embolism.

## Case presentation

A 69-year-old man with a history of primary lung adenocarcinoma and previous surgical treatment (video-assisted thoracoscopic left lower lobectomy associated with mediastinal lymph node dissection), presented for percutaneous lung biopsy after two nodules were identified in a follow-up thoracic CT (right upper and lower lobes).

The procedure was performed under local anesthesia. An 18-gauge core biopsy set was used to access the right upper lobe nodule in a single shot without complications. Immediately after the biopsy needle removal the patient transiently lost consciousness, became hemodynamically unstable, and was transferred to the emergency room (ER). In the ER he was hypotensive (arterial blood pressure of 72/42 mmHg), had a cardiac pulse of 45 bpm, and presented new symptoms of left-sided hemiparesis with a Glasgow Coma Scale (GCS) of 14.

**Figure 1:**
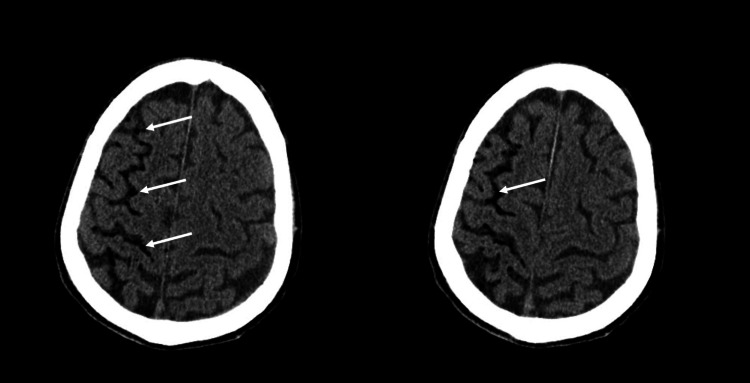
Cerebral CT performed prior to hyperbaric treatment. The white arrows highlight the air emboli.

A cerebral CT scan was performed, revealing multiple air emboli in the right cerebral sulcus (Figure 1) and the thoracic CT showed old known lesions without any acute complications of the biopsy.

Normobaric supplemental oxygen with a high fraction of inspired oxygen (FiO2 100%) with a non-rebreather mask and a fluid challenge (500 mL of crystalloid for 15 minutes) was administrated and the patient was positioned in the Trendelenburg position with right lateral decubitus. The patient was referred for emergent hyperbaric oxygen treatment and transferred to our hospital. Upon arrival, he was breathing spontaneously and was hemodynamical stable, with a GCS of 14. On neurological examination, he showed left-sided hemiparesis with paresthesias of the left upper limb and showed left hemispatial neglect. Diagnosis of cerebral air arterial embolism with less than four hours of evolution was confirmed.

**Figure 2:**
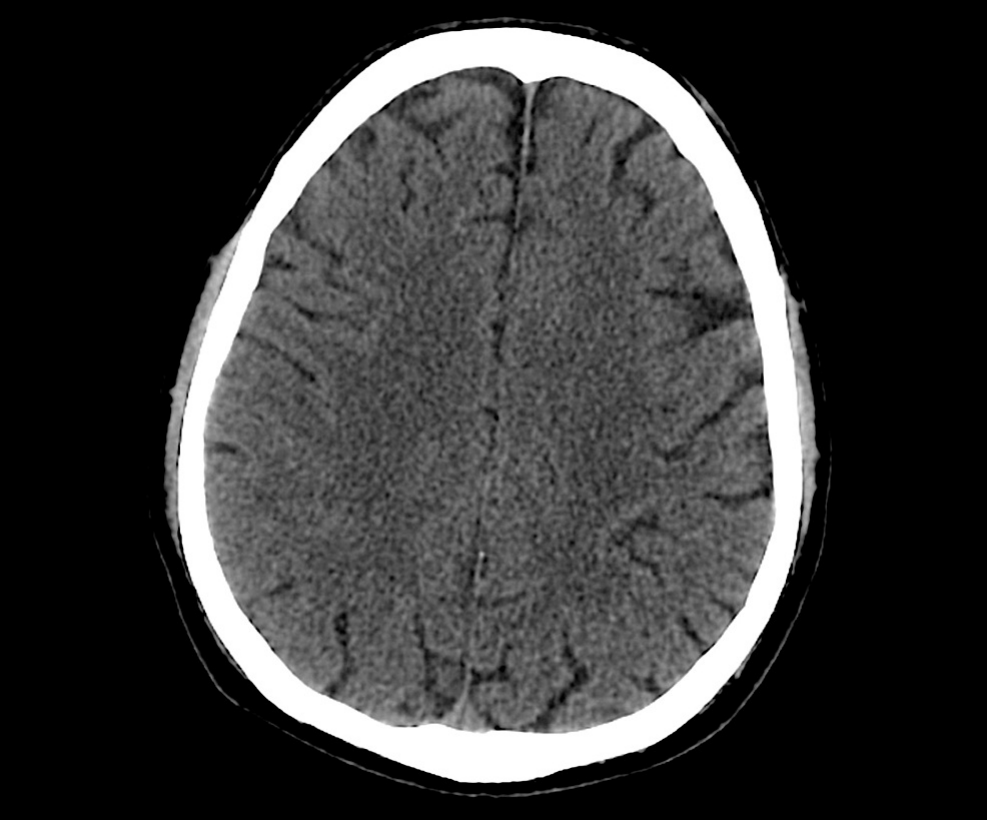
Cerebral computerized tomography scan following hyperbaric treatment with complete resolution of air embolism.

Hyperbaric oxygen treatment with US Navy Treatment Table 6 with two extensions of 20 minutes at 18 meters (2.8 Atmosphere Absolute) was performed. An initial bolus of 1.5 mg/kg of lidocaine followed by a perfusion at 1 mg/kg/h was administered. The patient completed a total duration of 345 minutes of treatment with an oronasal mask. The hyperbaric session occurred without any complications and by the end of the session, the neurological deficits had significantly improved with the left inferior limb fully recovered and the upper left limb maintaining a decreased ability to extend (scored 4/5 on the Medical Research Council scale). A cerebral CT scan performed at the end of the treatment showed complete resolution of air embolism (Figure 2).

The patient was transferred to a referral hospital the next day with complete recovery. 

## Discussion

The entry of gas into pulmonary circulation or arteries of systemic circulation may lead to systemic arterial air embolism. Common mechanisms of gas entry into circulation include an open pulmonary vein to the atmosphere directly through a biopsy needle when the stylet is removed, the needle itself may promote a fistulae formation between pulmonary veins and alveoli if it penetrates an alveolar space and air may be directly introduced into arterial circulation which then reaches pulmonary veins through pulmonary vasculature. After air entrance factors such as cough, Valsalva maneuvers, and obstructive pulmonary disease with air trapping can increase the pressure gradient between the airway and pulmonary veins promoting air entrance and air embolus [2,3].

Air embolism diagnosis is based on clinical findings and a high level of suspicion is necessary in order not to delay appropriate treatment [2]. Deterioration of cardiovascular, respiratory, and neurologic systems are the main sequelae of vascular embolism, and symptoms depend on infusion rate and volume injected [5]. The final diagnosis is made with a CT scan where air bubbles can be visualized either in the cerebral territory, in the aorta, or in intracardiac and pulmonary territories [2].

Immediate management of air embolism is the prevention of further air entrance and hemodynamic support [6]. Hyperbaric oxygen is the first-line treatment for gas embolism. Breathing 100% oxygen at a pressure above atmospheric pressure not only decreases the bubble size but also promotes nitrogen resorption from the bubble, leading to the resolution of the embolism [7]. According to Boyle’s law, when a hyperbaric environment is administered compression of bubbles and volume reduction occurs. In fact, at 3 atmospheres absolute (ATA), the bubble is reduced by one-third leading to better elimination and restored circulation. Under hyperbaric conditions, oxygen partial pressure in plasma reaches higher levels than those under atmospheric pressure and tissue hyperoxia has been demonstrated to decrease cerebral edema [8]. US Navy treatment tables set the exact times and depths that the patient should be treated in the hyperbaric chamber. Table 6 is considered the standard of care for arterial air embolism. It consists of a five-minute compression phase at 18 meters of seawater (MSW) depth under 100% oxygen followed by four oxygen cycles of 20 minutes each with short air intervals. The decompression phase is divided into two oxygen cycles of 60 minutes each at about 9 msw until a return to surface pressure occurs. An extension of the table is used when symptoms remain unchanged by the end of the second oxygen cycle at 18 msw [9]. In our case, two extensions were used due to the persistence of severe symptoms within the first 20 minutes.

Early recognition of air embolism and the beginning of hyperbaric oxygen therapy up to six hours after the beginning of symptoms is essential for patients with cerebral air embolism [3]. Patients that start hyperbaric treatment less than six hours after the beginning of symptoms have better outcomes than those undergoing treatment later [10]. In this case report diagnosis, referral, and beginning of treatment occurred within four hours which probably contributed to the good neurological outcome.

Although hyperbaric oxygen remains the first line treatment in air embolism, lidocaine has been studied as a drug capable of improving neurologic outcomes in such patients. Lidocaine is neuroprotective in arterial gas embolism as it reduces associated intracranial hypertension, lowers neuronal metabolism, preserves nerve conduction, and is a potent anti-inflammatory drug [11]. A bolus followed by lidocaine perfusion was used in our patient following this rationale.

## Conclusions

Although transthoracic lung biopsy is a routinely performed procedure, complications such as gas embolism can occur. Despite its rarity, arterial air embolism is dangerous and can be fatal. Rapid identification of symptoms can improve morbimortality in these patients, therefore, physicians who perform lung biopsies should be aware of this complication as the time taken for referral to hyperbaric treatment will dictate the prognosis of neurologic deficits.
